# Apolipoprotein-A-I for severe COVID-19-induced hyperinflammatory states: A prospective case study

**DOI:** 10.3389/fphar.2022.936659

**Published:** 2022-09-26

**Authors:** Stanislas Faguer, Arnaud Del Bello, Chloé Danet, Yves Renaudineau, Jacques Izopet, Nassim Kamar

**Affiliations:** ^1^ Referral Center for Rare Kidney Diseases, Department of Nephrology and Organ Transplantation, University Hospital of Toulouse, Toulouse, France; ^2^ Faculty of Medicine, University Paul Sabatier—Toulouse 3, Toulouse, France; ^3^ French National Institute of Health and Medical Research, U1297 (Institute of Metabolic and Cardiovascular Diseases), Toulouse, France; ^4^ Department of Clinical Pharmacy, University Hospital of Toulouse, Toulouse, France; ^5^ French National Institute of Health and Medical Research, U1291 (INFINITY), Toulouse, France; ^6^ Laboratory of Immunology, University Hospital of Toulouse, Toulouse, France; ^7^ Laboratory of Virology, University Hospital of Toulouse, Toulouse, France

**Keywords:** apolipoprotein-A-I, ApoA-I, HDL, cytokine storm, COVID-19, inflammation, endothelium

## Abstract

Viral infections can promote cytokine storm and multiorgan failure in individuals with an underlying immunosuppression or specific genetic background. Hyperinflammatory states, including critical forms of COVID-19, are characterized by a remodeling of the lipid profile including a dramatic decrease of the serum levels of apolipoprotein-A-I (ApoA-I), a protein known for its capacity to reduce systemic and lung inflammation, modulate innate and adaptive immunity, and prevent endothelial dysfunction and blood coagulation. In this study, four immunocompromised patients with severe COVID-19 cytokine storm that progressed despite standard-of-care therapy [Omicron (*n* = 3) and Delta (*n* = 1) variants] received 2– 4 infusions (10 mg/kg) of CER-001, an ApoA-I-containing HDL mimetic. Injections were well-tolerated with no serious adverse events. Three patients treated while not on mechanical ventilation had early clinical and biological improvement (oxygen withdrawal and correction of hematological and inflammatory parameters, including serum levels of interleukin-8) and were discharged from the hospital 3–4 days after CER-001 infusions. In the fourth patient who received CER-001 after orotracheal intubation for acute respiratory distress syndrome, infusions were followed by transient respiratory improvement before secondary worsening related to ventilation-associated pneumonia. This pilot uncontrolled exploratory compassionate study provides initial safety and proof-of-concept data from patients with a COVID-19 cytokine storm receiving ApoA-I. Further randomized controlled trial evaluation is now required to ascertain whether ApoA-I has any beneficial effects on patients with a COVID-19 cytokine storm.

## Introduction

Severe SARS-CoV-2-associated diseases (COVID-19) are characterized by acute respiratory distress syndrome (ARDS) with local and systemic inflammation; complement activation; infiltrating neutrophils, monocytes, and macrophages; and pulmonary microangiopathy with fibrin thrombi and activated platelets (Kessel et al., 2021). In fatal cases, autopsies demonstrated the development of significant vasculopathy and increased vascular congestion in the lungs (Villalba et al., 2022). In addition, extensive analyses of COVID-19 ARDS identified phenotypes and molecular changes that distinguish it from other causes of ARDS (Empson et al., 2022). In rare cases, a COVID-19-associated cytokine storm may culminate in a hyperinflammatory state similar to, but still distinct from, autoinflammatory macrophage activation syndrome (Kessel et al., 2021).

COVID-19 evolved as a biphasic disorder including first underlying inborn or acquired immunodeficiency (type I/III-interferon response deficiency) leading to viral escape to immune defenses, and then a hyperinflammatory response promoting lung injury, endotheliopathy, and coagulopathy (Asano et al., 2021; Carapito et al., 2021; Paludan and Mogensen, 2022). Among other features, critical COVID-19 is characterized by high circulating levels of interleukin (IL)-1Ra, IL-6, IL-8, TNF-α, and ICAM-1 and low levels of FasL (Kessel et al., 2021; del Valle et al., 2020). This finding prompted the use of immunomodulatory approaches (e.g., corticosteroids or IL-1, IL-6, or JAK inhibitors) to prevent or treat COVID-19 ARDS and secondary lung fibrosis and ultimately to prevent refractory respiratory failure (van de Veerdonk et al., 2022). These treatments, used alone or in combination, are associated with some degree of improvements but have unpredictable effects. In addition, they may be associated with an increased risk of secondary infection, including life-threatening opportunistic infections mitigating their positive effects (Gangneux et al., 2022). This is particularly true in individuals with underlying immunosuppression, like those who received solid organ transplantation.

Targeted and unbiased metabolomic approaches have identified low serum levels of high-density lipoprotein (HDL cholesterol) and apolipoprotein-A-I (ApoA-I) as strong predictive factors of severe forms of COVID-19 (Sun et al., 2020; Begue et al., 2021; Hilser et al., 2021). Alterations of the lipoprotein plasma composition were demonstrated (for instance, downregulation of apolipoproteins, clusterin, and paraoxonase) (Begue et al., 2021). In addition, SARS-CoV-2 infection also triggers a humoral response against ApoA-I that may modulate the outcome of COVID-19 (Pagano et al., 2021). Interestingly, several lines of evidence point to the role of ApoA-I in modulating inflammation and innate and adaptive immunity in various settings, suggesting a potential therapeutic use in COVID-19 (Sorokin et al., 2020). Therefore, ApoA-I can inhibit the activity of monocytes (Hyka et al., 2001; Smythies et al., 2010), inhibit the cross talk between dendritic cells and natural killer cells in terms of IL-12 and IFN-γ (Kim et al., 2005), decrease T-lymphocyte activation by activated macrophages (Hyka et al., 2001), and reduce the inflammatory response of type II pneumocytes to a viral challenge (van Lenten et al., 2004). In mice, an ApoA-I mimetic reduced the severity of influenza-related pneumonia (van Lenten et al., 2002). Very recently, Kelesidis et al. (2021) reported that the ApoA-I mimetic peptide 4F attenuates *in vitro* the replication of SARS-CoV-2 and associated apoptosis, oxidative stress, and inflammation (IL-6 production) in epithelial cells.

CER-001 is an engineered, pre-β HDL particle that contains human recombinant ApoA-1 together with natural phospholipids, combined into a single small discoidal particle (Keyserling et al., 2017). CER-001 was formerly developed for secondary prevention of cardiovascular diseases, but we recently reported that it can be proposed in patients with inherited lecithin-cholesterol-acyl-transferase deficiency to prevent kidney disease progression (Faguer et al., 2021). A preliminary study by Tanaka et al. (2022) reported a good tolerance of CER-001 in a critically ill patient with severe COVID-19 and secondary infection and a reduction in inflammatory markers. In addition, native HDL may exert a potent antiviral effect against SARS-Cov-2 (Cho et al., 2021). Altogether, these findings put forward the hypothesis that ApoA-I supplementation may prevent the progression of COVID-19 toward critical forms and/or reverse the cytokine storm induced by the SARS-CoV-2 infection.

In this study, we aimed to assess the tolerance of ApoA-I supplementation in four critically ill individuals who developed a COVID-19-associated hyperinflammatory state and were included in an ApoA-I compassionate-access program. As a secondary objective, their outcomes were reported.

## Patients and methods

### Approval and patients’ inclusion

The four patients included in this study were treated at the University Hospital of Toulouse (France) under a compassionate use program that was approved by the French Agency for the Safety of Drugs and Health Products (Agence Française de Sécurité du Médicament et des Produits de Santé [ANSM]; authorization #2021-104249; #2022-107250; #2022-108293; #2022-108518). All patients provided informed consent to receive CER-001 and to be included in the Nephrogene Biobank which was approved by the French National Ethical Review Board (DC-2011-1388). The study was conducted from 15 December 2021 to 31 January 2022. Patients had documented COVID-19 (nasopharyngeal PCR) and hyperinflammatory state characterized by a serum level of ferritin higher than 1000 μg/L and inflammation-related organ injury (respiratory failure, coma, cytopenia, and hepatitis). They were also characterized by an ApoA-I serum level below 0.9 g/L (normal value > 1.1 g/L).

### Drugs delivery

CER-001 was generously offered by Abionyx Pharma. Abionyx Pharma had no access to the data during the treatment period and did not participate in the article writing. Standard-of-care treatment was pursued, as appropriate.

### Clinical follow-up

Patients were followed up throughout their hospitalization stay and underwent physical examinations and routine blood sampling (complete blood count, arterial blood gases, inflammatory markers, kidney and liver functions, and lipid tests). Cytokine measurements (IL-1β, IL-6, IL-8, and TNF-α) were performed immediately before and at several time points after the first administration of CER-001, using the ELLA nanofluidic system (Bio-Techne, France). Clinical and biological characteristics prior to and following CER-001 administration were compared descriptively.

The major endpoints were survival and adverse events (safety part of the study). The secondary endpoints were the length of hospitalization, the evolution of inflammatory parameters (ferritin and cytokines), and the oxygen supports in patients with ARDS.

## Results

### Cases histories

*Patient 1* was a 52-year-old male patient with a history of IgA vasculitis, diabetes mellitus, and ischemic heart disease and had received a kidney transplant in 2018. He had been given three doses of mRNA vaccine but developed only weak anti-SARS-CoV-2 immunity (anti-spike antibodies 15.5 BAU/mL). He developed symptoms of COVID-19 on 17 December 2021 (fever, diarrhea, and dyspnea) and was admitted to the hospital on December 25. Oxygen saturation was 92% in room air, and oxygen supplementation was started (1 L/min). The chest CT scan showed a bilateral interstitial lung disease compatible with COVID-19 (parenchyma extension 25%). Nasopharyngeal PCR identified SARS-CoV-2 (variant-of-concern (VOC) Delta). Blood tests showed a hyperinflammatory state (ferritin 5,037 μg/L and C-reactive protein 34 mg/L), liver test abnormalities (AST and ALT 2.5 and 3.5 times the upper limit normal (ULN) values, respectively), and thrombocytopenia. Tacrolimus was pursued, mycophenolate mofetil was withdrawn, and dexamethasone was introduced (6 mg/day) with antibiotics. On day 2, the blood tests showed pancytopenia and progression of the hyperinflammatory state (ferritin 6,870 μg/L and C-reactive protein 55 mg/L). He received one infusion of the monoclonal anti-IL-6R antibody tocilizumab (8 mg/kg i.v.) and one infusion of neutralizing monoclonal anti-SARS-Cov-2 antibodies (casirimivab/imdevimab). On day 4, ferritin increased to 19,219 μg/L, AST and ALT increased to 17 and 14 times the ULN values, respectively, and arterial lactates were at 2.7 mmol/L. Bone marrow aspirate showed features of hemophagocytosis. Blood PCR of SARS-CoV-2 was weakly positive. Worsening hypoxia required increased oxygenation (4 L/min; PaO_2_ 62 mmHg), and the CT scan showed progressive lung lesions typical of COVID-19 (50% of the parenchyma). Despite increasing dexamethasone to 10 mg/day, serum triglycerides and ferritin increased to 3.2 mmol/L and 27,394 μg/L, respectively, on day 6.

*Patient 2* was a 38-year-old female patient with a history of systemic lupus erythematosus and being overweight and had received a kidney transplant in 2011. She had been given three doses of mRNA vaccine but developed no anti-SARS-CoV-2 immunity. She developed symptoms of COVID-19 on 4 January 2022 (cough, chills, diarrhea, and fever) and was admitted to the transplantation ward on January 14. Nasopharyngeal PCR identified SARS-CoV-2 (VOC Omicron). Upon admission, SaO_2_ was 94% while receiving 9 L/min of oxygen with a facial mask. The chest CT scan showed typical lesions of COVID-19 (extension 50%). Blood tests showed hepatitis with cytolysis and cholestasis (7–10 times the ULN values, respectively), acute kidney injury (KDIGO stage 1), and hyperinflammation (ferritin 2,000 μg/L and C-reactive protein 107 mg/L). High-flow oxygen supplementation, awake prone position, dexamethasone (10 mg/day), tocilizumab (8 mg/kg once), and antibiotics were started. Everolimus was withdrawn, and tacrolimus was pursued. On day 4, despite full-code therapy, high-flow oxygen supplementation was still required, and the hyperinflammatory state worsened (ferritin 2,800 μg/L).

*Patient 3* was a 47-year-old female patient with a history of diabetes mellitus, adrenal Cushing’s syndrome, hypertension, and end-stage kidney disease requiring chronic kidney replacement therapy since 2020. She did not receive anti-SARS-CoV-2 vaccination and had no anti-SARS-CoV-2 immunity at the time of admission to the hospital. She developed symptoms of COVID-19 on 15 January 2022 (cough, dyspnea, abdominal pain, and fever) and was admitted to the hospital on January 19. Nasopharyngeal PCR identified SARS-CoV-2 (VOC Omicron). The chest CT scan showed mild to moderate lung lesions typical of COVID-19 (10%–25%). She did not require oxygen supplementation. Blood tests showed hyperinflammatory syndrome (ferritin 4,350 μg/L and C-reactive protein 55 mg/L) with a moderate increase in AST and ALT (2 and 1.5 times the ULN values, respectively) and mild thrombocytopenia and anemia. Dexamethasone (6 mg/day) was introduced. On day 3, hyperferritinemia (4,142 μg/L) and liver test abnormalities persisted, and she developed encephalopathy leading to admission to the intensive care unit.

*Patient 4* was a 59-year-old male patient with a history of hepatitis B, liver transplantation in 2006, HHV8-negative Kaposi’s sarcoma (complete remission), and end-stage kidney disease requiring chronic kidney replacement therapy since 2020. He had received three doses of mRNA vaccines but developed no anti-SARS-CoV-2 antibodies. Owing to familial exposure to SARS-CoV-2, nasopharyngeal PCR was performed on 6 January 2022, identifying the VOC Omicron variant. He developed symptoms of COVID-19 on January 15 (asthenia) but had no respiratory symptoms. The chest CT scan showed mild to moderate lung lesions typical of COVID-19 (10%–25%). On January 17, dyspnea, cough, and fever developed. Upon admission, PaO_2_ was 54 mmHg in room air, his respiratory rate was 30 cycles/min, and body temperature was 38.5°C. Blood tests showed hyperferritinemia (1,223 μg/L) and leukopenia (1,080 cells/mm^3^). A CT scan showed progression of lung lesions (25%–50%). Oxygen supplementation, dexamethasone (10 mg/day), tocilizumab (8 mg/kg, once), antibiotics, and fresh frozen plasma from convalescent patients were given. Mycophenolate mofetil was withdrawn, and tacrolimus was pursued. On day 5, acute respiratory failure developed requiring orotracheal intubation and mechanical ventilation with neuromuscular blocking. Blood tests showed a hyperinflammatory state (ferritin 4,535 μg/L) with increased AST and ALT (three times the ULN values). At that time, the bronchoalveolar fluid culture was negative, suggestive of critical COVID-19 only. The PaO_2_ to FiO_2_ ratio was in the range of 150–180. Antibiotics were pursued.

### Dosing

According to the available information regarding its safety and pharmacokinetic/pharmacodynamic (Keyserling et al., 2017), CER-001 was given intravenously over 0.5–1 h at a dose of 10 mg/kg at hours 0 and 12 (patient 1) and hours 0, 12, 24, and 48 (patients 2, 3, and 4). In order to address the safety of the procedure, patient 1 received only two infusions of CER-001, whereas patients 2–4 received four infusions. Administration was preceded by anti-histaminic prophylaxis with hydroxyzine (50 mg i.v.). In all patients, dexamethasone was pursued.

### General safety

Patients 1, 2, and 3 did not develop any serious adverse events. Patient 4 developed two episodes of ventilation-associated pneumonia (VAP; *Klebsiella pneumoniae* and *Aspergillus fumigatus* plus mucormycosis) and one bacteremia (*Staphylococcus haemolyticus*).

### Bioefficacy: lipid profiles

**FIGURE 1:**
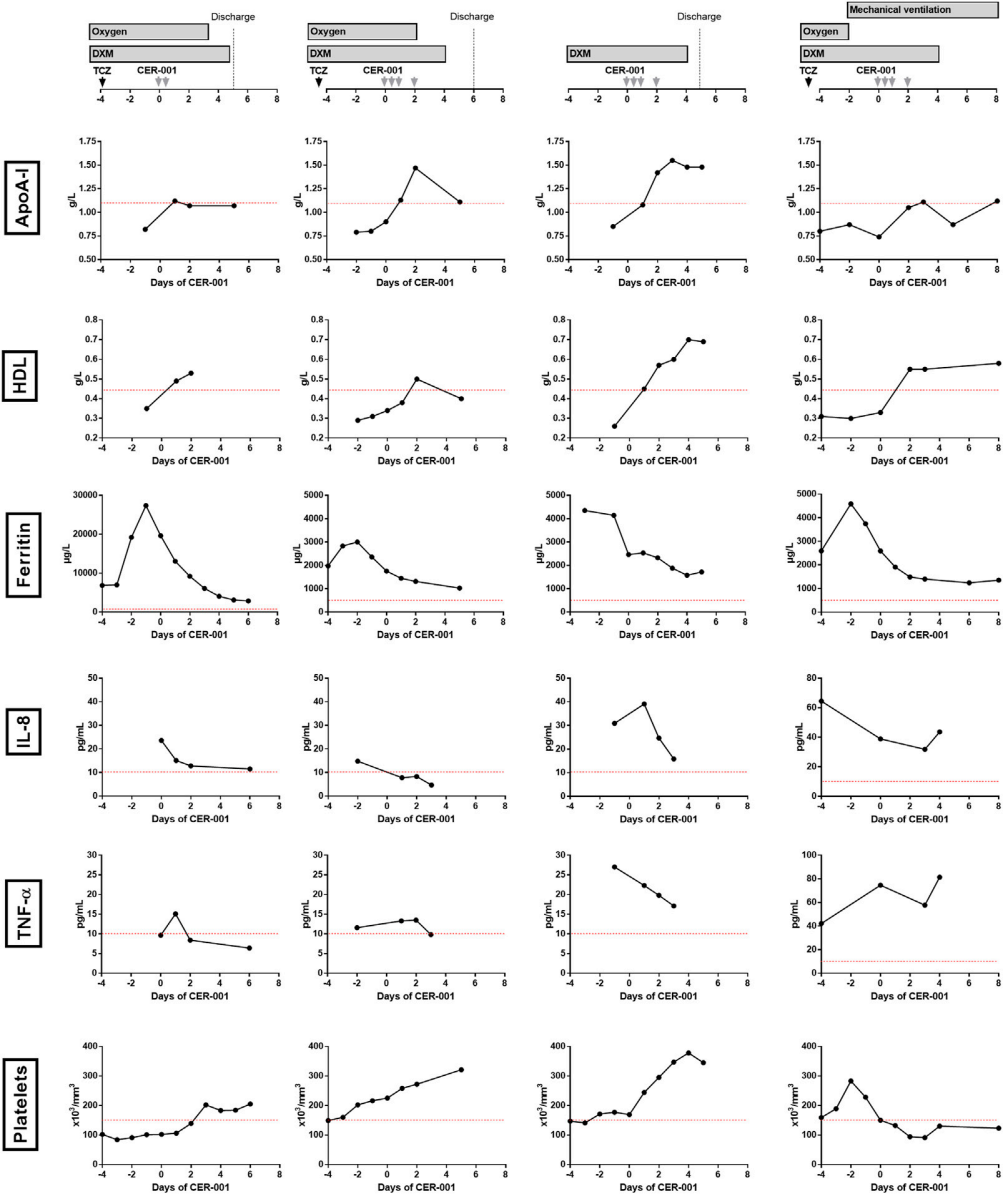
Outcomes of four individuals with the COVID-19-associated cytokine storm who received CER-001 as salvage therapy. DXM, dexamethasone; TCZ, tocilizumab; ApoA-I, apolipoprotein-A-I; IL-8, interleukin-8; TNF-α, tumor necrosis factor-α; IL-6, inteleukin-6.

As shown in Figure 1, all four patients had very low serum levels of ApoA-I (range 0.74–0.79 mg/L, normal value > 1.1 g/L) and HDL (range 0.26–0.35, normal value > 0.45 g/L) and high serum levels of triglycerides (range 2.16–3.4 g/L, normal value < 1.5 g/L) when CER-001 was started. Lipid tests were not available at the time of admission to the hospital. Following infusion of CER-001, ApoA-I and HDL levels normalized in all patients at day 2 but remained in the lower range of the normal values in most inflammatory patients. In patient 4, who developed ventilator-associated pneumonia 3 days after the start of CER-001, ApoA-I subsequently decreased below the normal values.

### Inflammation kinetic

At baseline, IL-1β was normal in all individuals, IL-6 was increased in the three patients who previously received tocilizumab and was normal in the latter (3.3–1,295 pg/ml), and TNF-α was moderately increased (9.7–42.1 pg/ml). IL-8 was the only inflammatory cytokine universally increased (>10 pg/ml; 14.8–64.5 pg/ml) (Figure 1). Following CER-001, IL-8 normalized in patients 1, 2, and 3. In patient 4, IL-8 decreased immediately after the injections and re-increased at the time of a ventilator-associated pneumonia. Serum levels of ferritin decreased from 6,616 ± 8,696 to 1,712 ± 815 μg/L, 6 days after the start of CER-001. The administration of anti-IL6R antibodies before CER-001 in 3 out of 4 patients precluded the analysis of C-reactive protein. Body temperature remained below 37.5° in all patients.

### Clinical outcomes

CER-001 administration was followed by a rapid improvement of the clinical condition of patients 1, 2, and 3, allowing them to be discharged from the hospital 3–4 days after the CER-001 infusions (Figure 1). In patients 1 and 2, oxygen supplementation was withdrawn 2 and 3 days after the administration, respectively. In patient 3, confusion resolved within 2 days. In these three patients, inflammatory parameters, liver tests, and blood cell count improved until discharge. Patient 4 received mechanical ventilation for 3 days when CER-001 was introduced. After a first phase of improvement (neuromuscular blocker withdrawal and sedation lightening) for 3 days, he secondarily developed several ventilator-associated pneumonia infections and ultimately died 1 month later. These infections were considered unrelated to the CER-001 treatment.

## Discussion

In this study, we reported the outcomes of four individuals with COVID-19-induced cytokine storm who received ApoA-I supplementation as salvage therapy. In addition to a very good acute tolerance of CER-001, as already observed in other settings outside critical care units (Nicholls et al., 2018; Faguer et al., 2021), we observed a rapid improvement in respiratory status, a decrease in inflammatory parameters, and the normalization of blood cell counts, paralleling the normalization of ApoA-I levels after CER-001 in 3 out of 4 patients. On the contrary, they developed critical COVID-19-related cytokine storm, and they could be discharged home, without oxygen support, as soon as 3–4 days after CER-001 infusions. After the completion of this compassionate-use access, Tanaka et al. (2022) also reported a case of severe COVID-19 in which CER-001 administration was followed by a dramatic decrease of inflammatory parameters during infusion. These preliminary clinical results and data obtained *in vitro* (Kelesidis et al., 2021) strongly support the development of a randomized double-blind clinical trial testing CER-001 in patients with hyperinflammatory COVID-19, especially in those more at risk of developing a critical disease.

Several studies have reported that ApoA-I and HDL-C are less abundant in COVID-19 patients (Poynard et al., 2020; Sun et al., 2020; Begue et al., 2021; Hilser et al., 2021), especially in the most severe forms, and that HDL-C from COVID-19 patients is less protective in endothelial cells submitted to inflammatory triggers and does not protect them from apoptosis (Begue et al., 2021). Low serum levels of ApoA-I may increase both the risk of developing COVID-19 (Poynard et al., 2020) and the risk of developing severe forms of COVID-19 (Begue et al., 2021). Such a decrease in ApoA-I or HDL-C is a common finding in cytokine storms and was observed in virus-induced and familial hemophagocytic lymphohistiocytosis (HLH) (Henter et al., 1991; Kraskovsky et al., 2021) and in dengue shock syndrome (Marin-Palma et al., 2019). Thus, the results derived from this study may potentially be valid in other settings of hyperinflammatory states. Beyond its potential ability to prevent severe forms of COVID-19, ApoA-I also modulates virologic control by hosts and their immune responses against various viruses (e.g., herpes simplex virus and dengue virus) (Srinivas et al., 1990; Coelho et al., 2021). Our data thus support future trials in other forms of virus-induced hyperinflammatory states, like viral HLHs or dengue hemorrhagic fever/shock syndrome.

Among the various inflammatory cytokines, IL-8 was the only cytokine that was universally increased (>10 pg/ml), confirming its ability to identify patients developing critical COVID-19 (Kessel et al., 2021). IL-6 was also increased though the previous use of tocilizumab which precluded firm conclusions (Nishimoto et al., 2008). Our proof-of-concept study was not designed to distinguish whether hyperinflammation and COVID-19 were indeed reversed by the ApoA-I supplementation itself or whether it was only a fortuitous association, but, following CER-001, we observed a rapid decrease in IL-8 in the three patients with a favorable outcome, paralleling the clinical and biological improvement. In patient 4, after a first phase of clinical improvement accompanied by ApoA-I normalization and IL-8 decrease, ventilation-associated pneumonia and clinical deterioration were accompanied by C-reactive protein and IL-8 increase and ApoA-I decrease.

In this study, patients received four infusions of CER-001 (10 mg/kg), but the use of a higher dose for the first injection (e.g., 15 mg/kg) may help reach the optimal concentrations of ApoA-I and non-oxidized HDL more rapidly to achieve maximal therapeutic effects. Also, the pharmacokinetic of CER-001 has only been described in healthy individuals (Keyserling et al., 2017). In a phase 1 clinical study, a 10 mg/kg dose led to an increase of ApoA-I in the range of 0.1–0.2 g/L in the first 12 h followed by rapid normalization (Keyserling et al., 2017) that prompted us to inject CER-001 at hours 0, 12, 24, and 48 to reach optimal values of blood ApoA-I in a setting of low baseline values. Whether its half-life is extended in critically ill patients with liver failure is currently unknown.

This study has several limitations including its uncontrolled design and the small size of the cohort which could be included in the compassionate access program. This did not allow us to identify COVID-19 patients with the highest probability to benefit from CER-001 and whether specific sub-phenotypes (according to the degree of inflammation, vasculopathy, or other parameters to be determined) may better respond to ApoA-1 supplementation. This will require additional analyses on larger cohorts. Second, one patient had a COVID-19 hyperinflammatory state but not respiratory failure. Our purpose was first to confirm the feasibility and tolerance of ApoA-I supplementation in the setting of critically ill COVID-19 patients. Here, we report the rapid improvement of some patients with COVID-19 ARDS or a hyperinflammatory state.

## Conclusion

In summary, this pilot uncontrolled exploratory compassionate study provides initial safety and proof-of-concept data from patients with a COVID-19 cytokine storm receiving ApoA-I. Further randomized controlled trial evaluation is now required to ascertain whether ApoA-I has any beneficial effects on patients with COVID-19 cytokine storm.

## Data Availability

The original contributions presented in the study are included in the article/Supplementary Material; further inquiries can be directed to the corresponding author.

## Abbreviations

ApoA-I, apolipoprotein-A-I; ARDS, acute respiratory distress syndrome; HDL, high-density lipoprotein; VOC, variant of concern.

## References

[B1] Asano T. Boisson B. Onodi F. Matuozzo D. Moncada-Velez M. Maglorius Renkilaraj M. R. L. (2021). X-linked recessive TLR7 deficiency in ∼1% of men under 60 years old with life-threatening COVID-19. Sci. Immunol. 6, eabl4348. 10.1126/sciimmunol.abl4348 34413140 PMC8532080

[B2] Begue F. Tanaka S. Mouktadi Z. Rondeau P. Veeren B. Diotel N. (2021). Altered high-density lipoprotein composition and functions during severe COVID-19. Sci. Rep. 11, 2291. 10.1038/s41598-021-81638-1 33504824 PMC7841145

[B3] Carapito R. Li R. Helms J. Carapito C. Gujja S. Rolli V. (2021). Identification of driver genes for critical forms of COVID-19 in a deeply phenotyped young patient cohort. Sci. Transl. Med. 14, eabj7521. 10.1126/scitranslmed.abj7521 34698500

[B4] Cho K. H. Kim J. R. Lee I. C. Kwon H. J. (2021). Native high-density lipoproteins (HDL) with higher paraoxonase exerts a potent antiviral effect against SARS-CoV-2 (COVID-19), while glycated HDL lost the antiviral activity. Antioxidants (Basel) 10, 209–211. 10.3390/antiox10020209 33535459 PMC7912765

[B5] Coelho D. R. Carneiro P. H. Mendes-Monteiro L. Conde J. N. Andrade I. Cao T. (2021). ApoA1 neutralizes proinflammatory effects of dengue virus NS1 protein and modulates viral immune evasion. J. Virol. 95, e0197420. 10.1128/JVI.01974-20 33827950 PMC8437349

[B6] del Valle D. M. Kim-Schulze S. Huang H. H. Beckmann N. D. Nirenberg S. Wang B. (2020). An inflammatory cytokine signature predicts COVID-19 severity and survival. Nat. Med. 26, 1636–1643. 10.1038/s41591-020-1051-9 32839624 PMC7869028

[B7] Empson S. Rogers A. J. Wilson J. G. (2022). COVID-19 acute respiratory distress syndrome: One pathogen, multiple phenotypes. Crit. Care Clin. 38, 505–519. 10.1016/j.ccc.2022.02.001 35667740 PMC9671407

[B8] Faguer S. Colombat M. Chauveau D. Bernadet-Monrozies P. Beq A. Delas A. (2021). Administration of the high-density lipoprotein mimetic CER-001 for inherited lecithin-cholesterol acyltransferase deficiency. Ann. Intern. Med. 174, 1022–1025. 10.7326/L20-1300 33646847

[B9] Gangneux J.-P. Dannaoui E. Fekkar A. Luyt C. E. Botterel F. De Prost N. (2022). Fungal infections in mechanically ventilated patients with COVID-19 during the first wave: the French multicentre MYCOVID study. Lancet. Respir. Med. 10, 180–190. 10.1016/S2213-2600(21)00442-2 34843666 PMC8626095

[B10] Henter J. I. Carlson L. A. Soder O. Nilsson-Ehle P. Elinder G. (1991). Lipoprotein alterations and plasma lipoprotein lipase reduction in familial hemophagocytic lymphohistiocytosis. Acta Paediatr. Scand. 80, 675–681. 10.1111/j.1651-2227.1991.tb11928.x 1867086

[B11] Hilser J. R. Han Y. Biswas S. Gukasyan J. Cai Z. Zhu R. (2021). Association of serum HDL-cholesterol and apolipoprotein A1 levels with risk of severe SARS-CoV-2 infection. J. Lipid Res. 62, 100061. 10.1016/j.jlr.2021.100061 33667465 PMC7923911

[B12] Hyka N. Dayer J. M. Modoux C. Kohno T. Edwards C. K. Roux-Lombard P. (2001). Apolipoprotein A-I inhibits the production of interleukin-1beta and tumor necrosis factor-alpha by blocking contact-mediated activation of monocytes by T lymphocytes. Blood 97, 2381–2389. 10.1182/blood.v97.8.2381 11290601

[B13] Kelesidis T. Madhav S. Petcherski A. Cristelle H. O'Connor E. Hultgren N. W. (2021). The ApoA-I mimetic peptide 4F attenuates *in vitro* replication of SARS-CoV-2, associated apoptosis, oxidative stress and inflammation in epithelial cells. Virulence 12, 2214–2227. 10.1080/21505594.2021.1964329 34494942 PMC8437485

[B14] Kessel C. Vollenberg R. Masjosthusmann K. Hinze C. Wittkowski H. Debaugnies F. (2021). Discrimination of COVID-19 from inflammation-induced cytokine storm syndromes using disease-related blood biomarkers. Arthritis Rheumatol. 73, 1791–1799. 10.1002/art.41763 33880885 PMC8251089

[B15] Keyserling C. H. Barbaras R. Benghozi R. (2017). Development of CER-001 : Preclinical dose selection through to phase I clinical findings. Clin. Drug Investig. 37, 483–491. 10.1007/s40261-017-0506-3 PMC5394142 28213743

[B16] Kim K. D. Lim H. Y. Lee H. G. Yoon D. Y. Choe Y. K. Choi I. (2005). Apolipoprotein A-I induces IL-10 and PGE2 production in human monocytes and inhibits dendritic cell differentiation and maturation. Biochem. Biophys. Res. Commun. 338, 1126–1136. 10.1016/j.bbrc.2005.10.065 16259956

[B17] Kraskovsky V. Harhay J. Mador M. J. (2021). Case of haemophagocytic lymphohistiocytosis following Epstein-Barr virus infection. BMJ Case Rep. 14, e241222. 10.1136/bcr-2020-241222 PMC8016084 33789863

[B18] Marin-Palma D. Sirois C. M. Urcuqui-Inchima S. Hernandez J. C. (2019). Inflammatory status and severity of disease in dengue patients are associated with lipoprotein alterations. PLoS ONE 14, e0214245. 10.1371/journal.pone.0214245 30901375 PMC6430398

[B19] Nicholls S. J. Andrews J. Kastelein J. J. P. Merkely B. Nissen S. E. Ray K. K. (2018). Effect of serial infusions of CER-001, a pre-β high-density lipoprotein mimetic, on coronary atherosclerosis in patients following acute coronary syndromes in the CER-001 atherosclerosis regression acute coronary syndrome trial: A randomized clinical trial. JAMA Cardiol. 3, 815–822. 10.1001/jamacardio.2018.2121 30046828 PMC6233644

[B20] Nishimoto N. Terao K. Mima T. Nakahara H. Takagi N. Kakehi T. (2008). Mechanisms and pathologic significances in increase in serum interleukin-6 (IL-6) and soluble IL-6 receptor after administration of an anti-IL-6 receptor antibody, tocilizumab, in patients with rheumatoid arthritis and Castleman disease. Blood 112, 3959–3964. 10.1182/blood-2008-05-155846 18784373

[B21] Pagano S. Yerly S. Meyer B. Juillard C. Suh N. Le Terrier C. (2021). SARS-CoV-2 infection as a trigger of humoral response against apolipoprotein A-1. Eur. J. Clin. Invest. 51, e13661. 10.1111/eci.13661 34324704 PMC8420318

[B22] Paludan S. R. Mogensen T. H. (2022). Innate immunological pathways in COVID-19 pathogenesis. Sci. Immunol. 7, eabm5505. 10.1126/sciimmunol.abm5505 34995097

[B23] Poynard T. Deckmyn O. Rudler M. Peta V. Ngo Y. Vautier M. (2020). Performance of serum apolipoprotein-A1 as a sentinel of Covid-19. PLoS One 15, e0242306. 10.1371/journal.pone.0242306 33216772 PMC7679025

[B24] Smythies L. E. White C. R. Maheshwari A. Palgunachari M. N. Anantharamaiah G. M. Chaddha M. (2010). Apolipoprotein A-I mimetic 4F alters the function of human monocyte-derived macrophages. Am. J. Physiol. Cell Physiol. 298, 1538–1548. 10.1152/ajpcell.00467.2009 PMC2889631 20219948

[B25] Sorokin A. v. Karathanasis S. K. Yang Z. H. Freeman L. Kotani K. Remaley A. T. (2020). COVID-19-Associated dyslipidemia: Implications for mechanism of impaired resolution and novel therapeutic approaches. FASEB J. 34, 9843–9853. 10.1096/fj.202001451 32588493 PMC7361619

[B26] Srinivas R. v. Birkedal B. Owens R. J. Anantharamaiah G. M. Segrest J. P. Compans R. W. (1990). Antiviral effects of apolipoprotein A-I and its synthetic amphipathic peptide analogs. Virology 176, 48–57. 10.1016/0042-6822(90)90229-k 2158697

[B27] Sun J. T. Chen Z. Nie P. Ge H. Shen L. Yang F. (2020). Lipid profile features and their associations with disease severity and mortality in patients with COVID-19. Front. Cardiovasc. Med. 7, 584987. 10.3389/fcvm.2020.584987 33344516 PMC7746652

[B28] Tanaka S. Begue F. Veeren B. Robert T. Faille D. Tashk P. (2022). First recombinant high-density lipoprotein particles administration in a severe ICU COVID-19 patient, a multi-omics exploratory investigation. Biomedicines 10, 754. 10.3390/biomedicines10040754 35453504 PMC9029957

[B29] van de Veerdonk F. L. Giamarellos-Bourboulis E. Pickkers P. Derde L. Leavis H. van Crevel R. (2022). A guide to immunotherapy for COVID-19. Nat. Med. 28, 39–50. 10.1038/s41591-021-01643-9 35064248

[B30] van Lenten B. J. Wagner A. C. Anantharamaiah G. M. Garber D. W. Fishbein M. C. Adhikary L. (2002). Influenza infection promotes macrophage traffic into arteries of mice that is prevented by D-4F, an apolipoprotein A-I mimetic peptide. Circulation 106, 1127–1132. 10.1161/01.cir.0000030182.35880.3e 12196340

[B31] van Lenten B. J. Wagner A. C. Navab M. Anantharamaiah G. M. Hui E. K. W. Nayak D. P. (2004). D-4F, an apolipoprotein A-I mimetic peptide, inhibits the inflammatory response induced by influenza A infection of human type II pneumocytes. Circulation 110, 3252–3258. 10.1161/01.CIR.0000147232.75456.B3 15533864

[B32] Villalba J. A. Hilburn C. F. Garlin M. A. Elliott G. A. Li Y. Kunitoki K. (2022). Vasculopathy and increased vascular congestion in fatal COVID-19 and ARDS. Am. J. Respir. Crit. Care Med.. Online ahead of Print. 10.1164/RCCM.202109-2150OC PMC9799276 35671465

